# Methods for safety and endpoint ascertainment: identification of adverse events through scrutiny of negatively adjudicated events

**DOI:** 10.1186/s13063-020-04254-w

**Published:** 2020-04-09

**Authors:** Alexander C. Fanaroff, Ghazala Haque, Betsy Thomas, Allegra E. Stone, Lynn M. Perkins, Matthew Wilson, W. Schuyler Jones, Chiara Melloni, Kenneth W. Mahaffey, Karen P. Alexander, Renato D. Lopes

**Affiliations:** 1 Cardiovascular Medicine Division, Penn Cardiovascular Outcomes, Quality and Evaluative Research Center, Leonard Davis Institute of Health Economics, University of Pennsylvania, Philadelphia, PA USA; 2 Duke Clinical Research Institute, Duke University, 200 Morris Street, Durham, NC USA; 3 Division of Cardiology, Duke University, Durham, NC USA; 4 Stanford Center for Clinical Research, Department of Medicine, Stanford Univeristy School of Medicine, Stanford, CA USA

## Abstract

**Background:**

The primary goal of phase 2 and 3 clinical trials is to evaluate the safety and effectiveness of therapeutic interventions, and efficient and reproducible ascertainment of important clinical events, either as clinical outcome events (COEs) or adverse events (AEs), is critical. Clinical outcomes require consistency and clinical judgment, so these events are often adjudicated centrally by clinical events classification (CEC) physician reviewers using standardized definitions. In contrast, AEs are reported by sites to the trial coordinating center based on common reporting criteria set by regulatory authorities and trial sponsors. These different requirements have led to the development of separate tracks for COE and AE review.

**Main body:**

Potential COEs that fail to meet standardized definitions for CEC adjudication – i.e. negatively adjudicated events (NAE) – may meet criteria for AEs. Trial oversight practices require the sponsor to process AEs regardless of how the AEs are submitted; therefore, review of NAEs may be necessary to ensure that important AEs do not go unreported. The Duke Clinical Research Institute (DCRI) developed and implemented a process for scrutinizing NAEs to detect potential missed serious AEs. Initial experience with this process across two trials suggests that approximately 0.2% of NAEs are serious unexpected AEs that were not otherwise reported and another 1.5% are serious expected AEs.

**Conclusions:**

Given their infrequent concealment of serious AEs in two large trials assessing cardiovascular outcomes, routine scrutiny of NAEs to identify AEs is not recommended at this time, though it may be useful in some trials and should be carefully considered by the trial team. Closer integration of data across safety surveillance and endpoint adjudication systems may enable scrutiny of NAEs when indicated while limiting complexity associated with this process.

## Background

Clinical trials conducted with the intent of earning regulatory approval for a new therapeutic intervention or a new indication for a previously-approved therapeutic intervention are required to demonstrate the product’s safety and effectiveness [1]. Trials evaluating interventions that seek to prevent the occurrence of clinical events accomplish this through the ascertainment of two major classes of events: (1) clinical outcome events (COEs), including events intended to be prevented by the investigational product and certain selected safety events (i.e. bleeding in a trial of an antithrombotic agent); and (2) adverse events (AEs), which represent off-target medical occurrences that may or may not be associated with the investigational product.

To reduce the potential for bias, in most cases, all suspected COEs should be adjudicated using standardized definitions by trained clinical events classification (CEC) reviewers blinded to treatment assignment. Although some controversy still exists about the need for CEC, with concerns centered around cost and the possibility that non-systematic or automated processes may provide a reasonable facsimile [2–4], CEC committees employing standardized definitions remain a key part of the gold standard process for systematic, precise, and reproducible event ascertainment and adjudication [5, 6]. In addition, sponsors, investigators, and regulators are responsible for protecting patients from being harmed by experimental products during the conduct of clinical trials. Study personnel and sponsors fulfill this responsibility by reporting certain types of AEs to experimental products to regulatory authorities promptly [7]. However, reactions to investigational products require knowledge of treatment assignment [8]. The need for both blinded physician adjudication of trial endpoint events and unblinded safety data reporting, a more rapid turnaround time for reporting safety events compared with COEs, and the need for committee review of COEs but not safety events, has therefore resulted in separate processes for COE adjudication and safety monitoring in most cases.

Phase 1 clinical trials, concerned exclusively with safety, often do not have a control group, so COEs and safety events are usually processed together. Phase 2 trials are nominally focused on safety; however, many Phase 2b trials also seek to evaluate efficacy or better characterize expected, on-target AEs. Phase 3 trials, often placebo-controlled, seek to establish an investigational product’s efficacy, and blinded COE adjudication is customary. Phase 2 and 3 trials often use separate teams for safety surveillance and COE adjudication. Furthermore, in late-phase clinical trials, most events that constitute COEs (such as death or myocardial infarction) may also meet criteria to be considered serious AEs (SAEs), which trigger mandatory reporting to regulatory authorities. However, since these events are associated with the disease being studied and are COEs, they are generally exempted from reporting as SAEs as long as there is not a suggested causal relationship between the treatment and the event, to avoid double reporting and reduce the burden of AE review [9]. Therefore, event processing varies across study phases, and the division between COEs and AEs becomes clearly demarcated in later phase studies.

Late-phase protocols provide sites with detailed instructions regarding which types of events to submit as potential trial endpoints and potential AEs. However, not all events submitted by sites as potential clinical endpoint events are ultimately adjudicated as clinical endpoint events. Some of these negatively adjudicated events (NAEs) may represent important AEs that should be reviewed and/or reported by a CEC coordinating center; however, no established protocol for the systematic, standardized evaluation of NAEs to detect AEs has been described. In the past, these NAEs have largely been taken at face value, but in reviewing recent clinical trials, the U.S. Food and Drug Administration (FDA) raised concerns about missed or hidden AEs in the pool of NAEs (personal communication and FDA trial audits). This led sponsors and researchers to consider how CEC and safety surveillance could be better integrated to scrutinize NAEs and address the FDA’s concerns. To the best of our knowledge, no published research has described methods for evaluating NAEs to uncover AEs. The purpose of this position paper is to describe and interpret our experiences with NAE evaluation for missed SAEs..

## Main text

### What is a NAE?

In multicenter randomized clinical trials (RCTs), potential COE data are entered in electronic case report forms, and supporting documentation may be forwarded to the coordinating center for adjudication. Trained physicians, comprising a CEC group, review the documents provided by the sites to determine whether a COE per standardized definitions has occurred. Standardized definitions ensure trial results are reliable and reproducible. Importantly, these standardized definitions include multiple separate criteria, often comprising elements of the history, physical examination, laboratory or imaging testing, and treatment. In order for a potential COE to be positively adjudicated (i.e. for the reviewer to indicate that the event occurred), multiple criteria often must be met.

**Fig. 1:**
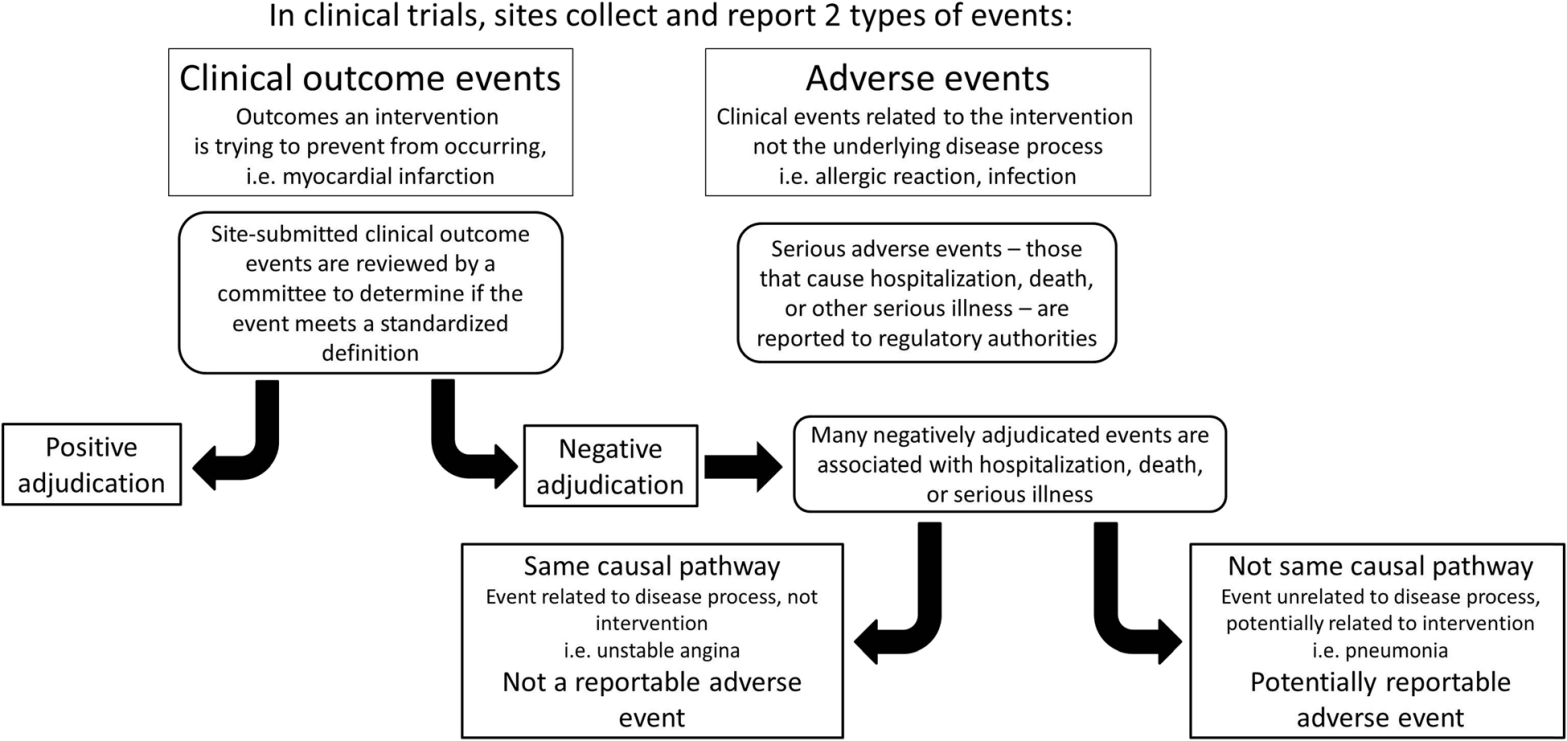
Potential concealment of AEs by NAEs. Negatively adjudicated events may conceal potentially reportable AEs if the event leads to hospitalization, death, or severe illness and is not attributable to the disease process the trial intervention is seeking to prevent. AE, adverse event; NAE, negatively adjudicated event

When a site-reported event does not meet all the criteria for positive adjudication by the CEC reviewers, then it is an NAE. NAEs occur for three reasons: (1) a COE did not occur, but an event representing a similar physiologic process did occur (i.e. unstable angina, rather than myocardial infarction); (2) a COE did not occur, but the event that did occur can be attributed to something else entirely (i.e. pneumonia leading to chest pain, rather than myocardial infarction); or (3) a COE likely occurred but documentation is inadequate to meet the protocol-specified definition (insufficient source documentation). Insufficient source documentation cases should be minimized by careful study planning and execution, but some number of these cases is likely unavoidable. Though all three of these represent NAEs, each has different implications for safety monitoring. In the second circumstance, the NAE is likely to represent an SAE that may be attributable to the intervention and require reporting, whereas in the first and third circumstances, the NAE is more likely to be attributable to the underlying disease process (Fig. 1).

### Why should NAEs be reviewed?

Sponsors, investigators, and regulators of clinical trials have a responsibility to protect participants enrolled in the study. FDA regulations and International Council for Harmonisation of Technical Requirements for Pharmaceuticals for Human Use Good Clinical Practice guidelines codify this responsibility by requiring sponsors to report AEs during trial conduct [7, 10]. Reporting requirements differ based on the severity and expectedness of AEs (Table 1). In general, SAEs require reporting when it appears that they occur more frequently in the treatment group than in the control group; every serious *and unexpected* adverse reaction (SUSAR) must be reported within 15 days of the sponsor’s discovery, 7 days if the event was fatal. In each case, reportable events require an association between the event and the investigational product, which requires review of unblinded data; the FDA specifically discourages sponsors from reporting blinded individual safety reports, which are uninformative and burden the system without benefit. COEs, by contrast, do not need to be reported to the FDA [7]; hence, sites do not submit potential COEs as AEs or SAEs.

**Table 1:** Adverse event definitions and reporting requirements

Event type	FDA definition	FDA reporting requirement
Adverse event (AE)	Any untoward medical occurrence associated with the use of a drug in humans, whether or not considered drug related	No specific reporting requirement
Serious adverse event (SAE)	AE that results in one of the following outcomes: death, threat to life, hospitalization or prolongation of an existing hospitalization, persistent incapacity or substantial disruption of the ability to conduct normal life functions, congenital anomaly, or important medical event (serious jeopardy of patient well-being requiring medical or surgical treatment in the judgment of the investigator)	When aggregate analysis of specific events observed in a clinical trial indicates that those events occur more frequently in the drug treatment group than in a concurrent or historical control group
Suspected unexpected serious adverse reaction (SUSAR)	SAEs that are unexpected prior to study conduct and likely to be caused by the investigational product	Each SUSAR must be reported within 15 days of the sponsor discovering it, and within 7 days if the event was life-threatening or fatal

The importance of identifying AEs, particularly SAEs, to protect patients during trial conduct provides the rationale for scrutinizing NAEs. There are numerous examples of phase 3 RCTs being stopped early to protect patient safety based on the accumulation of SAEs [10, 11], highlighting the importance of idenfiying these events. One recent trial stopped for accumulation of SAEs is the REGULATE-PCI clinical trial (Effect of the REG1 anticoagulation system versus bivalirudin on outcomes after percutaneous coronary intervention), which was stopped after 3232 of an anticipated 13,200 patients were enrolled due to an unexpectedly imbalanced number of allergic reactions in patients assigned to REG1 compared with placebo, including one fatal event [12].

The process of surveying for missed COEs from reported safety events is longstanding, with many clinical trial coordinating centers employing programs to trigger review of relevant safety terminology to look for missed COEs. Review of NAEs represents this process in reverse. Previously, when CECs determined that a site-reported COE did not meet CEC criteria for positive adjudication, these NAEs were often not further evaluated in any systematic way. In many cases, sites report hospitalizations separately as SAEs, but not in all cases (i.e., when sites determine the hospitalization was for a COE). Therefore, NAEs may conceal important AEs or SAEs, and events could be missed. For the most part, AEs concealed by NAEs will meet criteria to be SAEs: In the Apixaban for Prevention of Acute Ischemic Events 2 (APPRAISE-2) trial, COE and safety event reporting were integrated, and site investigators prospectively reported whether potential COEs met criteria for seriousness (i.e. death, hospitalization, prolongation of hospitalization, etc.). In that study, 63% of NAEs met criteria to be considered SAEs; the proportion of these events that were not reported by sites as AEs was not reported [13]. If these NAEs were not simultaneously reported as both potential clinical outcome events and AEs (which is not desirable), then, without systematic review of NAEs, they may have represented missed SAEs. If, for example, the CEC reviewers determine that a site-reported heart failure (HF) hospitalization in a patient taking an investigational product was not due to HF but was instead due to pneumonia, and if more patients taking the investigational product than would be expected are diagnosed with pneumonia, then the higher incidence of pneumonia would be important to track as it may require reporting.

### Findings from systematic scrutiny of NAEs in two trials

The simplest method for identifying NAEs that may represent unreported AE/SAEs would be to manually cross-check every NAE against the trial’s reported AEs. For NAEs not reported as AEs, further medical review would determine whether the NAE represents a potential SAE However, approximately 25% of site-reported COEs are negatively adjudicated by the CEC [14–16], and manual cross-checking and medical review of hundreds or thousands of NAEs would increase trial cost and complexity, a particular concern in the current era [17]. The ultimate purpose of scrutinizing NAEs is to capture unreported SAEs and SUSARs. Thus, the goal of any system of NAE review should be to reduce manual cross-checking and central review while still ensuring adequate review. The CEC group already reviews the events which are NAEs, so leveraging this process makes sense.

**Fig. 2:**
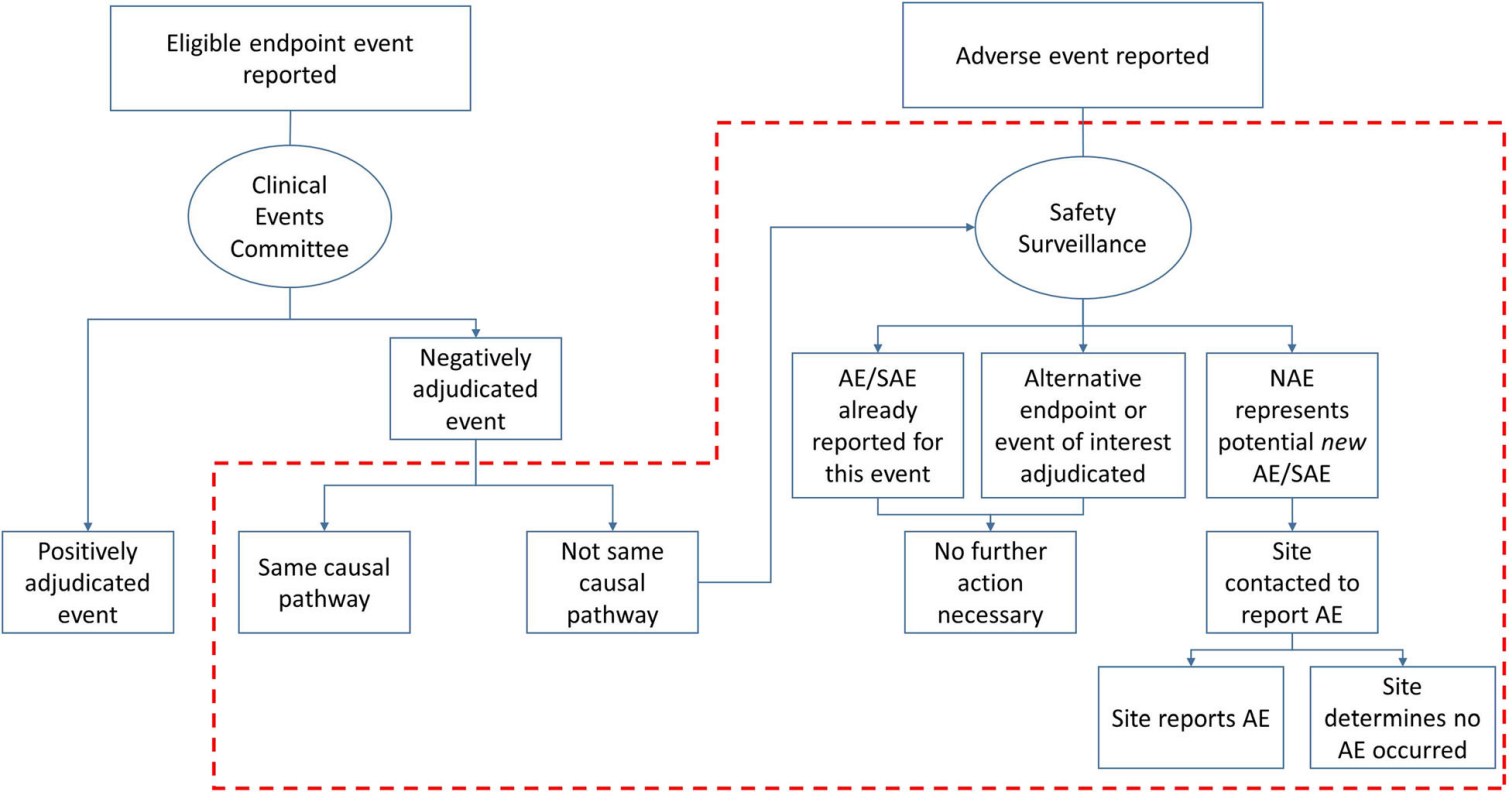
Pathway for NAE review. Area outlined in *red dashed box* represents DCRI system for managing NAEs; without implementation of this system, NAEs receive no further consideration and some AEs/SAEs could be missed. AE, adverse event; DCRI, Duke Clinical Research Institute; NAE, negatively adjudicated event; SAE, serious adverse event

The Duke Clinical Research Institute (DCRI) CEC group has developed a process for scrutinizing NAEs, which it has implemented in two clinical trials (Fig. 2). The crux of the process is the concept of whether the NAE has the *same causal pathway* as the potential COE. For each NAE, CEC reviewers indicate whether the event, often a hospitalization encounter, was caused by the same or similar pathophysiologic process as the site-reported or triggered potential COE, or whether it was caused by an alternative pathophysiologic process, representing a reportable safety event.. For example, if a site reports, as a potential COE, that a patient was hospitalized with a myocardial infarction (MI), but the rise and fall in cardiac biomarkers is insufficient for diagnosis of MI per the standardized definition, and the CEC reviewer determines the correct diagnosis was unstable angina, this represents the same causal pathway as MI. Similarly, when a potential COE is negatively adjudicated due to insufficient documentation, or the admission is for chest pain without another diagnosis, this NAE represents the same causal pathway as the COE. In most cases, same causal pathway events are related to the underlying disease (or concern for the same process) rather than the investigational product. If this is the case, they would not represent potential unidentified SAEs and would not require further review. An NAE may also not require further review if it meets criteria for another COE in the trial (e.g., if the potential COE does not meet criteria for MI but does meet criteria for HF hospitalization or cardiovascular death). In this case, the NAE is not actually an NAE, since it meets criteria for a different COE.

By contrast, in a case where a site reports a HF hospitalization as a potential COE, but the reviewers determine that the true etiology of the event was pneumonia, this event represents an alternative pathophysiologic process. If the true etiology of the event is unclear, this represents an unclear causal pathway. These alternative and unclear causal pathway events represent situations where a potential SAE may be the cause of the NAE, and these cases are referred to the coordinating center’s safety surveillance team.

The DCRI Safety Surveillance group (SSG) manually cross-checks the AE/SAE database for an event related to each NAE. If an SAE corresponding to that hospitalization has already been reported by the site, no further action is taken. If no AE has been reported corresponding to this NAE, then the SSG reviews the associated source documents for potential SAEs. If a potential SAE is identified, the SSG contacts the site and requests that the investigators consider reporting the event as an SAE. The site then determines whether an SAE occurred, and reports accordingly. If a site refuses to document a potential SAE, then the sponsor documents “event disputed.”

This process has been implemented in two recently completed clinical trials enrolling a total of 24,215 patients (Table 2). Both trials assessed the effect of a pharmacologic agent on cardiovascular events, and clinical outcome endpoints for both trials were death, MI, stroke, hospitalization or revascularization for unstable angina, and hospitalization for heart failure. In trial A, of 1774 NAEs (26.2% of all site-reported potential COEs), 606 (34.1%) represented an alternative or unclear causal pathway, but only 2 (0.1%) resulted in previously unreported SAEs being reported, as most NAEs had already been reported as an SAE by the site. In trial B, of 1208 NAEs (44.1% of all site-reported potential COEs), 718 (59.4%) were due to an alternative or unclear causal pathway, and 20 (1.6%) resulted in previously unreported SAEs being reported, including two SUSARs (0.2%). Thus, in these two trials, < 2% of NAEs were ultimately determined to be SAEs unreported by sites prior to manual review of NAEs.

**Table 2:** Results of NAE review in 2 completed clinical trials

Event category	Number of events (% of NAEs)	
	Trial A (*N* = 1774 NAEs; 26.2% of site-reported COEs)	Trial B (*N* = 1208 NAEs; 44.1% of site-reported COEs)
Same causal pathway^a^	1168 (65.8%)	490 (40.6%)
Not same causal pathway^a^	606 (34.2%)	718 (59.4%)
AE/SAE already reported	118 (6.7%)	517 (42.8%)
Alternative COE	369 (20.8%)	71 (5.9%)
Potential new AE	119 (6.7%)	130 (10.8%)
New AE reported	2 (0.1%)	20 (1.7%)
SUSAR	0 (0.0%)	2 (0.2%)

^a^ Causal pathway designation determined by CEC adjudicator based on whether the NAE was caused by the same or similar pathophysiologic process as the site-reported potential clinical outcome event (i.e. myocardial infarction and unstable angina), or whether it was caused by an alternative pathophysiologic process. *AE* adverse event, *COE* clinical outcome event, *NAE* negatively adjudicated event, *SAE* serious adverse event, *SUSAR* suspected unexpected serious adverse reaction

### Critical considerations of this process

In our early experience, NAEs infrequently represent unreported SAEs, and this review process may increase coordinating center workload and cost. First, the reviewers are asked to perform another step in adjudication. Same/alternative causal pathway concepts may also be challenging to explain to reviewers and may increase training duration. In addition, same/alternative causal pathway concepts are difficult to define in a standardized manner. For example, it is unclear whether hospitalization for drainage of a symptomatic pleural effusion falls under the same pathophysiologic process as a HF hospitalization, especially if key data regarding the nature of the effusion are not reported. This limitation is inescapable, although it can be mitigated by training reviewers to better understand the event adjudication and safety surveillance processes. Lastly, each of the alternative/unclear causal pathway NAEs need to be manually cross-checked against the reported AE/SAE data, creating an additional task for the SSG.

Ultimately, this closer integration of CEC and safety surveillance systems would allow automated cross-checking of NAE and SAE data. One potential solution, employed in APPRAISE-2, is to design primary data collection to capture potential clinical endpoints on both SAE and clinical endpoint forms; however, this process substantially increases the burden on participating sites and may still require manual cross-checking of SAE and clinical endpoint events to avoid duplicate SAE reporting [13].

Alternatively, the NAE process would benefit from smarter case report form design. Rather than asking sites to report potential clinical endpoint events and SAEs separately or in duplicate, trials could ask sites to report all potential events through a uniform event reporting system, and whether the site considers the event to meet criteria for “seriousness” could be the first question asked by the event reporting system. The second question could ask sites whether the event represents a potential COE or AE. Serious events would require review of NAEs to look for SAEs, and non-serious events would not. Regardless, each event would have a unique identifier that would allow the coordinating center to track the event through the adjudication and/or safety monitoring process in an automated way. The requirement that each event is uniquely categorized removes redundancy and adds a built-in check that prevents SAEs from going unreported. In pragmatic clinical trials, which may employ streamlined or automated event adjudication or safety monitoring protocols [17, 18], hospitalizations identified by billing records or patient report could be evaluated algorithmically in a similar manner. The exact details of this process for any given study will need to be right-sized for the investigational product, program, and clinical trial, but the common denominator is enhanced communication between safety surveillance and CEC groups.

Regardless of the ultimate strategy employed to track NAEs and capture unreported SAEs, this process is likely to add at least some complexity to clinical trial conduct. Our experience indicates that this process may be relatively low-yield with only two SUSARs identified in two trials enrolling > 24,000 patients. As NAEs continue to be tracked and reviewed in ongoing clinical trials, the rate at which this process yields SAEs and SUSARs should be monitored. As more data about the costs and benefits of NAE review accrue, it should be a focus of ongoing dialogue between regulators, academia, and industry. In late-phase, post-approval studies of drugs with well-established safety profiles, scrutiny of NAEs may not be necessary at all, provided adequate systems are in place for monitoring drug safety using real-world observational data.

## Conclusions

Scrutiny of NAEs in phase 2 and 3 clinical trials enables detection of potentially unreported AEs. In our experience, however, this process is very low yield, introduces greater complexity. We do not recommend routine scrutiny of NAEs to detect unreported AEs in cardiovascular outcome trials, though it may be useful in some contexts and should be carefully considered by trialists during the trial planning phase. Efforts to develop systems to seamlessly integrate data across safety surveillance and endpoint adjudication systems may reduce cost and complexity while enabling scrutiny of NAEs when appropriate.

## Data Availability

The data that support the findings of this study are available from the Duke Clinical Research Institute but restrictions apply to the availability of these data, which were used under license for the current study, and so are not publicly available. Data are however available from the authors upon reasonable request and with permission of relevant trial sponsors.

## Abbreviations

AE: Adverse event
APRAISE-2: Apixaban for Prevention of Acute Ischemic Events 2
CEC: Clinical events classification
COE: Clinical outcome event
DCRI: Duke Clinical Research Institute
FDA: Food and Drug Administration
HF: Heart failure
MI: Myocardial infarction
NAE: Negatively adjudicated event
RCT: Randomized controlled trial
REGULATE-PCI: Effect of the REG1 anticoagulation system versus bivalirudin on outcomes after percutaneous coronary intervention
SAE: Serious adverse event
SSG: Safety Surveillance Group
SUSAR: Suspected unexpected serious adverse reaction
